# Compliance Disengagement in Research: Development and Validation of a New Measure

**DOI:** 10.1007/s11948-015-9681-x

**Published:** 2015-07-15

**Authors:** James M. DuBois, John T. Chibnall, John Gibbs

**Affiliations:** 1 Division of General Medical Sciences, School of Medicine, Washington University in St. Louis, Campus Box 8005, 4523 Clayton Avenue, St. Louis, MO 63110 USA; 2 Saint Louis University, St. Louis, MO USA; 3 Ohio State University, Columbus, OH USA

**Keywords:** How I Think Questionnaire, Research compliance, Moral disengagement, Ethical decision-making

## Abstract

In the world of research, compliance with research regulations is not the same as ethics, but it is closely related. One could say that compliance is how most societies with advanced research programs operationalize many ethical obligations. This paper reports on the development of the How I Think about Research (HIT-Res) questionnaire, which is an adaptation of the How I Think (HIT) questionnaire that examines the use of cognitive distortions to justify antisocial behaviors. Such an adaptation was justified based on a review of the literature on mechanisms of moral disengagement and self-serving biases, which are used by individuals with normal personalities in a variety of contexts, including research. The HIT-Res adapts all items to refer to matters of research compliance and integrity rather than antisocial behaviors. The HIT-Res was administered as part of a battery of tests to 300 researchers and trainees funded by the US National Institutes of Health. The HIT-Res demonstrated excellent reliability (Cronbach’s alpha = .92). Construct validity was established by the correlation of the HIT-Res with measures of moral disengagement (*r* = .75), cynicism (*r* = .51), and professional decision-making in research (*r* = −.36). The HIT-Res will enrich the set of assessment tools available to instructors in the responsible conduct of research and to researchers who seek to understand the factors that influence research integrity.

## Introduction

This paper describes the rationale for developing a measure of compliance disengagement in research (the How I Think about Research measure), the process of developing the measure, and a study involving 300 researchers funded by the US National Institutes of Health (NIH) to test the validity of the new measure.

### Context

In the world of research, compliance with research regulations is not the same as ethics, but it is closely related. One could say that compliance is how most societies with advanced research programs operationalize many ethical obligations. US federal regulations for the protection of human subjects make this explicit: The “common rule” for human subjects protection is viewed as a specification of the Belmont principles of respect for persons, beneficence, and justice (National Commission 1979; Office of Human Research Protections 2009). Similarly, research ethicists recognize responsibilities to care for animals, to respect the privacy of health information, and to cite articles when using excerpts. All of these responsibilities have been translated into research regulations, and ethics textbooks routinely discuss ethical and regulatory obligations side by side (Levine 1986; Shamoo and Resnik 2015; Emanuel et al. 2003).

### The Rationale for Compliance

The rationale for compliance pertains to accountability, protection from harm, and appropriate balance or reduction of self-serving biases. The American Association for the Advancement of Science (2015) reports that the US spent nearly $63 billion on nondefense research and development in 2014. Burk (1995) has suggested that the “expansion of actionable misconduct beyond the bounds of outright fraud should not be surprising, and may be inevitable … Where public monies are used, there must be public accountability” (p. 340).

Research regulations serve multiple functions. In most instances, regulations resulted from failures in the responsible conduct of research; they were efforts to compel, for example, the protection of human subjects, proper care and use of animals, or the integrity of data (Rollin 2006; Jones 1993; National Bioethics Advisory Commission 2001).

While researchers may appreciate a certain amount of latitude in making decisions about research design, regulations also serve to provide guidance where expectations were once vague (Steneck 2007). Research ethics is rarely about choosing good over evil, but rather about balancing competing good aims, e.g., balancing access to data or biospecimens with respect for the persons who provided the data or biospecimens. Regulations can provide guidance on how to strike the balance or on what processes to use when making such decisions.

Finally, regulations and compliance systems can serve to reduce the influence of bias. Violations of the responsible conduct of research sometimes are intentional, but at other times are not. A growing body of literature indicates that self-serving bias operates below the threshold of awareness and influences professional decisions (AAMC-AAU 2008; Moore and Loewenstein 2004; Irwin 2009). Compliance and oversight programs can play an important role in ensuring that research is conducted with integrity even when researchers might have powerful subliminal motives to cut corners.

Compliance should be part and parcel of quality research. To the extent that compliance can promote the aim of good research, one could say that compliance is a virtue of research professionals (DuBois 2004). It is simply part of being an effective researcher.

### The Burden of Compliance

At the same time, the burden of compliance has grown significantly over the past three decades. It is not uncommon for an academic institution in the United States to have written policies and require training of selected personnel in 10 or more domains, including animal welfare, conflicts of interest, controlled substances, effort reporting, export control, Health Insurance Portability and Accountability Act’s (HIPAA) privacy rule, human research protections, intellectual property, and research integrity. The National Council of University Research Administrators publishes a book, *Research and compliance*, that “distills essential information from mounds of federal laws, regulations and circulars, covering more than 100 of the most significant sets of requirements referenced in federal contracts and grants” (Youngers and Webb 2014, back cover).

The resulting burdens of compliance can slow and discourage research:

The past two decades have witnessed increasing recognition that the administrative workload placed on federally funded researchers at U.S. institutions is interfering with the conduct of science in a form and to an extent substantially out of proportion to the well-justified need to ensure accountability, transparency and safety. (National Science Board 2014, p. 1)

Principal investigators report spending 42 % of their grant-funded time on administrative tasks (National Science Board 2014). A report from the National Research Council asserted that the problem of excessive regulatory burdens is expected to cost universities “billions of dollars over the next decade.” (National Research Council 2012, p. 16) Moreover, institutional policies sometimes appear to have a primary intention of protecting institutions rather than protecting the integrity of science or the welfare of human and animal subjects (Koski 2003).

Finally, compliance can have an unintended side effect. Ethical obligations to society, to human or animal subjects, or to scientific peers may be reduced to a ritual performance aimed at satisfying a regulatory requirement. For example, the obligation to obtain informed consent—which should involve presentation of information and ascertaining understanding and voluntary agreement, with follow-up over time—can be reduced to “consenting someone,” which means obtaining a signature on a consent form, that is, doing the minimum needed to satisfy requirements. And when compliance requirements appear unjust or unreasonable, researchers may bypass the system and conduct research without oversight (Keith-Spiegel and Koocher 2005; Martinson et al. 2006). The Federation of Societies for Experimental Biology’s 2013 survey on administrative burden (N = 1324 individuals, mostly principal investigators) found that “One common perception of regulatory oversight among responders was that regulations ‘punished all’ for the ‘mistakes of a few’” (Federation of American Societies for Experimental Biology 2013). Thus, it is not difficult to understand how some researchers may disengage from compliance—that is, they may rationalize spending less time on compliance than necessary or avoiding some domains of compliance altogether.

### The Problem of Noncompliance

We explained above that there are good reasons for compliance requirements, and good reasons why they can be perceived as problematic. Nevertheless, noncompliance causes significant problems for institutions, investigators, subjects and science. For institutions, noncompliance can involve spending enormous amounts of time and money on investigations, paying fines, and having research programs suspended. Institutions invest heavily in compliance education because federal sentencing guidelines for institutions provide reduced penalties for institutions that have an effective and vital compliance program, including effective compliance training (Grant et al. 1999; Olson 2010). For investigators, noncompliance can lead to suspension of protocols, loss of research privileges, loss of privileges to obtain government funding, and prohibitions from publishing data (Neely et al. 2014). For human subjects, noncompliance may involve failures of informed consent, privacy protection, or safety monitoring (National Bioethics Advisory Commission 2001). For science, noncompliance can contribute to bad publicity for the field, diminished public trust, and the publication of questionable research data (Irwin 2009).

### Why Does Noncompliance Occur?

Despite the problems that noncompliance causes, research-intensive universities conduct an estimated 2–3 investigations of serious noncompliance each year involving violations of human subjects protections, research integrity, animal care, or conflict of interest policies (DuBois et al. 2013a). A meta-analysis of self-report surveys found that 2 % of investigators admitted to engaging at least once in research misconduct defined as plagiarism or data fabrication or falsification (Fanelli 2009).

Why do researchers fail to comply with regulations and policies? Many different answers are plausible. In a literature review, DuBois, Anderson et al. identified 10 environmental factors that are hypothesized to contribute to professional wrongdoing by providing a motive, means, or opportunity (DuBois et al. 2012). Factors included financial rewards, lack of oversight, ambiguous norms, vulnerable victims, and playing conflicting roles. However, when the same research group later examined 40 cases of actual research misconduct, they found that few environmental factors characterized the cases. The most common characteristic of cases was self-centered thinking, which was mentioned in 48 % of cases (DuBois et al. 2013b).

Drawing from their experience on review boards, Neely et al. (2014) state that the cause of noncompliance can be “that an investigator is overloaded, does not know the regulations, or does not take the time to pay attention to the details” (p. 716). However, each of these “causes” begs a question. Overload leads to prioritization—why is compliance given low priority? Why does the principal investigator not know the regulations? When researchers lack knowledge of technical matters they frequently turn to colleagues or the literature to find answers—why do they not do the same with questions about compliance? Why is the investigator not taking time to pay attention to the details? Do they pay attention to details of their data analysis or their research budgets? Explanations that focus on the demands of the research environment (Martinson et al. 2009)—the pressure to publish and obtain external research funding—similarly lead us to ask, using the logic of Samenow’s (2001) exploration of criminal behavior, why most researchers behave with integrity in the face of the same pressures.

Based on the experience of two authors over the past 3 years delivering remediation training to investigators who were referred for noncompliance, we hypothesize that many instances of noncompliance occur when researchers use cognitive distortions that support disengagement from compliance. We propose this as an extension of moral disengagement theory.

### Cognitive Distortions that Support Moral Disengagement

Most of the literature on moral disengagement has focused on antisocial behavior such as theft and violent crime. Central to moral disengagement theory is the idea that human beings tend to use cognitive strategies to protect a self-identity as a decent person. “People do not ordinarily engage in reprehensible conduct until they have justified to themselves the rightness of their actions. What is culpable can be made righteous through cognitive reconstrual” (Bandura et al. 1996, p. 365).

In describing the self-concept of convicted criminals who engaged in antisocial behaviors, Samenow (2001) observes:

The antisocial person regards himself as a good human being. He may admit momentarily to having done something wrong, especially if he believes it will be to his advantage. … But if one were to inquire whether, deep down, he regards himself as a bad person, the answer would be in the negative. As one man remarked, ‘If I thought of myself as evil, I couldn’t live.’ (p. 297)

Mechanisms of moral disengagement permit this self-identity to persist despite antisocial behaviors. In a longitudinal study of youths considered at risk for antisocial behavior, Hyde, Shaw, et al. found that moral disengagement was more strongly correlated with the development of antisocial behavior than any environmental variables. Moral disengagement further served as a moderator variable that explained whether neighborhood, rejecting parenting, and lack of empathy would predict antisocial behavior (Hyde et al. 2010).

This same psychological dynamic appears to characterize “normal” (as opposed to antisocial) levels of dishonesty. After conducting a series of six experiments on dishonest behavior, Mazar et al. (2008) conclude that “people who think highly of themselves in terms of honesty make use of various mechanisms that allow them to engage in a limited amount of dishonesty while retaining positive views of themselves” (p. 642). One of the primary mechanisms they use is characterization—changing the way they characterize their dishonesty. This fits well with Bandura’s work on moral disengagement, which involves the use of justification strategies such as using euphemistic labels or advantageous comparisons to prevent self-sanctioning (Bandura et al. 1996; Bandura 1999).

In an analysis of cases of research misconduct that involved coding of statements by the wrongdoers and cluster analysis, Davis et al. (2007) found that rationalizations comprised 2 of 7 clusters. Rationalizations included statements that clearly minimized the harmfulness of misconduct, blamed others, or assumed the worst if they did not fabricate data or plagiarize. This fits well with the growing body of literature cited above that indicates that self-serving bias characterizes the decision-making of normal individuals and professionals (AAMC-AAU 2008; Moore and Loewenstein 2004). It would appear that rationalization is not just for antisocial personalities, and self-serving bias is not just for narcissists (although it is heightened in these clinical populations).

## The How I Think Questionnaire

The How I Think (HIT) Questionnaire was developed to assess self-serving cognitive distortions particularly in adolescent populations with antisocial tendencies or behaviors (Barriga et al. 2001). The HIT questionnaire focuses on four cognitive distortions, that is, four thinking errors that distort the interpretation of a situation in favor of self-interests. The four cognitive distortions are assuming the worst, blaming others, minimizing/mislabeling, and self-centered thinking. Table 1 provides a definition of each of the cognitive distortions and presents a HIT item that represents each. All of the HIT’s cognitive distortion items are written with reference to one or another of four behaviors that are representative of delinquent behavior: oppositional-defiance, physical aggression, lying, and stealing. The HIT also includes anomalous responding (AR) items, which serve as a built-in measure of socially desirable responding, and positive filler (PF) items which serve to reduce the questionnaire’s emphasis on negative behaviors, perhaps making its purpose less transparent. The HIT questionnaire underwent modification and improvement during the course of its development. The original version included 52 cognitive distortion items and 8 AR items. Item analyses of development sample data utilized selection criteria such as criterion group discrimination and correlation with antisocial behavior measures. The final version comprised 54 items (39 cognitive distortion, 8 AR items, and 7 PF items).

**Table 1:** Adaptation of the How I Think (HIT) Questionnaire into the How I Think about Research (HIT-Res) Questionnaire

Construct	Definition	Sample original HIT item	Behavioral referent	Sample HIT-Res item	Behavioral referent
*Four self*-*serving cognitive distortions*					
Assuming the worst	Attributing bad intentions to others or focusing on worst-case-scenario as if it cannot be avoided	I might as well lie–when I tell the truth, people don’t believe me anyway	Lying	Consent forms don’t protect participants because no one reads them anyway	Protections (animal and human)
Blaming others	Misattributing blame to others or a temporary state (e.g., I was in a bad mood)	If someone leaves a car unlocked, they are asking to have it stolen	Stealing	The pressure to get grants almost forces people to take liberties with their data	Research integrity
Minimizing/Mislabeling	Denying that misbehavior causes harm or is wrong, or dehumanizing victims	Everybody breaks the law, it’s no big deal	Oppositional defiance	Everybody has conflicts of interest, it’s no big deal	Conflicts of interest
Self-centered thinking	Focusing on one’s own views and needs to the exclusion of the legitimate views and needs of others	When I get mad, I don’t care who gets hurt	Physical aggression	I know which corners I can cut to meet a deadline	General responsible conduct of research
Anomalous responding	Responses that are socially desirable but unlikely to be sincere	I have sometimes said something bad about a friend	n/a	I have sometimes said something bad about a colleague	n/a
Positive filler	Items that are unscored but serve to reduce the focus on negative behaviors and attitudes	When friends need you, you should be there for them	n/a	When trainees need you, you should be there for them	n/a

The HIT consists of a series of statements that are rated on a 1–6 Likert-type scale ranging from (1) strongly disagree to (6) strongly agree without a “neutral” option. The overall HIT score consists of the mean value of responses to items representing any of the four cognitive distortions, thus allowing for a range from 1 to 6, with higher scores indicating higher usage of self-serving cognitive distortions. Wallinius et al. (2011) found a mean score of 1.88 (SD = .46) in a sample of adult non-offenders and 2.72 (SD = .90) among adult offenders.

A recent meta-analysis of studies conducted with 29 independent samples (*N* = 8186) found that the HIT has demonstrated high levels of reliability and validity. Internal consistency reliability (Cronbach’s alpha) was excellent across populations, with a mean alpha of .93, 95 % CI [.92, .94] (Gini and Pozzoli 2013). The validity of the HIT has been supported in relation to numerous constructs, including the ability to distinguish delinquent from non-delinquent populations, and highly significant (*p* < .001) positive correlations with measures of externalizing behavior (*r* = .52), aggressive behavior (*r* = .38), antisocial behavior (*r* = .55), delinquent behavior (*r* = .41), and low empathy (*r* = .42) (Gini and Pozzoli 2013).

Wallinius, Johansson et al. found similar results from their administration of the HIT to adult and adolescent offenders and non-offenders in Sweden (Wallinius et al. 2011). Across all four samples the HIT demonstrated excellent reliability (alpha = .90 to .96). Among both adult and adolescent samples, the HIT identified significantly higher levels of self-serving cognitive distortions among offenders. The study by Wallinius et al. is notable for our purposes because it supported the validity and reliability of the HIT with adults, with non-offenders, and with both males and females.

We considered alternatives to adapting the HIT. For example, Medeiros et al. (2014) developed a taxonomy of biases in ethical decision-making. While it is useful as a research framework, we rejected it as a framework for assessment because we felt (a) most of the self-serving cognitive biases they identified could be subsumed under one of the four distortions operationalized by the HIT-Res (e.g., “abdication of responsibility,” “diffusion of responsibility,” and “unquestioning deference to authority” are all forms of “blaming others” as we operationalized the concept); (b) Bandura found that the biases that support moral disengagement are not multifactorial; and (c) a simpler framework is advantageous from a pedagogical perspective. While they examine additional “biases”—such as moral insensitivity and changing norms—these are quite different from self-serving biases and require different measurement approaches.

In what follows we report on the initial psychometric evaluation of the HIT-Res in a sample of researchers funded by NIH.

## Methods

### Adapting the HIT

Given that the HIT has demonstrated reliability and validity in identifying self-serving cognitive distortions that play a role in perpetuating deviant behavior, and that recent data suggest that cognitive distortions are used by ordinary people who engage in milder forms of wrongdoing (such as displaying “normal” levels of dishonesty), we decided to adapt the HIT for use with researchers. We maintained a focus on the original four cognitive distortions, but changed the four behavioral referents to matters of research compliance: conflicts of interest (COI); human and animal subject protections (HSP/ASP); research misconduct (RM: falsification, fabrication, and plagiarism); and the general responsible conduct of research (RCR). To be clear, our primary focus in developing the HIT-Res was the use of cognitive distortions—not the behavioral referents, which were meant simply to increase the ecological validity of the test. As we developed behavioral referents, the purpose was to provide research-specific examples of the use of four cognitive distortions; no attempt was made to address all matters of research compliance and integrity, and no plan was made to analyze the behavioral referents as distinct constructs.

The HIT includes 39 cognitive distortion items (11 assuming the worst, 10 blaming others, 9 minimization/mislabeling, and 9 self-centered thinking), 8 anomalous responding items, and 7 positive filler items. Under the direction and review of the original author of the HIT, item content was adapted to the research context by the first two authors (both of whom have extensive research and assessment experience with research ethics and psychological constructs), with the goal of staying as close as possible to the original HIT item wording (see Table 1) and maintaining a balance of the 4 behavioral referents within each of the 4 cognitive distortions. Items were also edited generally to reduce ambiguity and promote equivalence of length. In rare cases, HIT items could not be converted to the research context and/or could not be linked to a research behavioral referent; in these cases, a HIT item was dropped and/or a new item was written to balance item content for a given cognitive distortion. These adaptations resulted in a set of 42 cognitive distortion items: 10 assuming the worst (3 RM, 3 RCR, 2 COI, 2 HSP/ASP), 12 blaming others (4 RCR, 3 RM, 3 HSP/ASP, 2 COI), 10 minimization/mislabeling (3 RM, 3 HSP/ASP, 3 COI, 1 RCR), and 10 self-centered thinking (3 RCR, 3 COI, 2 RM, 2 HSP/ASP); 7 anomalous responding items; and 6 positive filler items. The reading level of this set of items was assessed using the Flesch–Kinkaid measure, which returned a reading level of grade 4.8, which is similar to the 4th grade reading level of the HIT (Barriga et al. 2001). This was considered advantageous given that, according to the National Science Foundation, nearly half of all post-doctoral trainees were born in non-native-English-speaking countries (National Science Foundation 2014). We named this set of 55 items the How I Think about Research (HIT-Res) questionnaire. We anticipated that HIT-Res might be shortened following testing by dropping items with low item-total correlations. Table 1 presents examples of original HIT and HIT-Res items in each domain.

### Participants and Recruitment

We recruited a convenience sample of 300 researchers who were funded by the NIH, working in the United States (US), and diverse in terms of career stage, age, and field of research. We built a recruitment database using the NIH RePORTER, an online database of all grants awarded by NIH, which can be sorted by funding mechanisms and identifies the principal investigator of each grant. In order to represent diverse career stages, we targeted individuals with two distinct kinds of funding: Training grants (T and K) and independent investigator grants (R01). In order to increase the number of eligible trainees, we also contacted the principal investigators of institutional research training programs funded through the Clinical and Translational Science Award (CTSA) program with the request that they share our recruitment email with their NIH-funded trainees.

From February through May 2014, potential participants were contacted by email with an invitation to participate in a study that aimed to evaluate a measure of how researchers make professional decisions. We estimated that participation would require 75–120 min to complete the full battery of measures and offered $100 in payment. If an individual did not complete the measures, reminder emails were sent at 1 and 4 weeks following initial contact. Each email contained a link to the online survey as well as a link to opt out of further contact.

### Survey Instrument: Platform, Measures, and Convergent Validity Hypotheses

The survey was conducted using Qualtrics survey software, which allows the use of many different response formats and provides HIPAA-compliant data security (www.qualtrics.com). We used the Qualtrics forced choice option and received complete data on the full battery of measures from 300 participants.

The survey included the following measures. Measures 2, 3, and 4 were used to assess convergent validity, measure 5 to assess concurrent criterion validity, and measure 6 to control for social desirability.

1. The HIT-Res, which was used for the first time in this validity study.
2. Propensity to Morally Disengage Scale (Moore et al. 2012). We used the 8-item version of the PMD (alpha reliability = .80), which consists of one item representing each of eight mechanisms of moral disengagement, such as euphemistic labeling and displacement of responsibility (e.g., “Some people have to be treated roughly because they lack feelings that can be hurt”). Moore et al. (2012) reported strong evidence for the convergent, discriminant, incremental, and predictive validity of the PMD. We expected the HIT-Res to be positively correlated with the PMD because they are both intended to measure the use of cognitive distortions or thinking errors that support moral disengagement. However, we also expected some divergence given that the HIT-Res assesses the use of cognitive distortions specifically with reference to research compliance, rather than general moral norms.
3. Global Cynicism Scale (GCS) (Turner and Valentine 2001). The GCS is an 11-item scale (alpha reliability = .86) that assesses level of cynicism (e.g., “When you come right down to it, it’s human nature never to do anything without an eye to one’s own profit”). Turner and Valentine (2001) reported compelling evidence for the convergent, discriminant, criterion-related, and nomological validity of the GCS. Cynicism has also been shown to be negatively correlated with good ethical decision making and positive organizational behavior (Mumford et al. 2006; Turner and Valentine 2001). We hypothesized that GCS scores would be positively correlated with the cognitive distortions assessed by the HIT-Res.
4. Narcissistic Personality Inventory (NPI-16) (Raskin and Terry 1988; Ames et al. 2006). The NPI-16 is a well-validated (convergent, discriminant, and predictive), reliable (alpha reliability = .72; retest reliability = .85), 16-item measure of narcissism (e.g., “I know that I am good because everybody keeps telling me so”). Previous studies have found that narcissism is negatively correlated with good ethical decision-making and positive organizational behavior (Mumford et al. 2006; Antes et al. 2007; Penny and Spector 2002). We expected that narcissism would be positively correlated with HIT-Res scores.
5. The Professional Decision-making in Research (PDR) measure (DuBois et al. 2015). The PDR is an adaptation of the Ethical Decision-Making Measure (EDM), which examines the use of good decision-making strategies when confronted with difficult decisions in research that defy one simple right answer (Mumford et al. 2006; Antes and DuBois 2014). In a validation study with 300 NIH-funded researchers, it demonstrated strong reliability (alpha reliability = .84; parallel forms reliability = .70) and construct validity (DuBois et al. 2015). Such strategies include seeking help, managing emotions, anticipating consequences, recognizing rules, and testing personal assumptions and motives. It consists of 16 vignette items presenting challenging situations; each item is followed by six response options (3 illustrate the use of good decision-making strategies, 3 violate one of more of the strategies) from which participants select the two they would be most likely to do if they were actually in the situation. One point is awarded when both options selected illustrate the use of good professional decision-making strategies. We hypothesized that higher scores on the HIT-Res would correlate with lower scores on the PDR, because compliance disengagement would decrease the use of strategies such as recognizing rules, considering consequences, and testing personal assumptions.
6. Marlowe–Crowne Social Desirability Scale (MCSDS) (Crowne and Marlowe 1960; Reynolds 1982). We used the 13-item form of the MCSDS (alpha reliability = .76) as a control variable to determine the extent to which responses on the HIT-Res might be determined by socially-desirable responding (e.g., “I have never been irked when people expressed ideas very different from my own”), and to examine convergent validity of the anomalous responding (AR) scale of the HIT-Res. Reynolds (1982) found strong evidence for convergent validity of the MCSDS and recommended the 13-item version as the best substitute for the original 33-item MCSDS.
7. A demographic survey that allowed us to describe our population and examine whether the HIT-Res correlates with variables such as gender, age, years of experience, field of study, and native language. See Table 2 for a description of demographic data collected.

**Table 2:** Demographics and differences among subgroups

Variable	N	Mean HIT-Res	SD	*F/t* value	*p* value
*Age*					
20–29	93	2.54	.55	F = 1.48	.22
30–39	134	2.50	.67		
40–49	52	2.46	.67		
>50	21	2.23	.51		
*Gender*					
Male	128	2.63	.70	t = 3.42	.001
Female	17	2.38	.55		
*Years doing research*					
0–5	104	2.53	.63	F = .92	.43
6–10	119	2.44	.60		
11–20	57	2.54	.69		
20+	20	2.35	.66		
*Funding status: trainee*					
Yes	152	2.53	.65	t = −1.36	.18
No	148	2.44	.61		
*Current research funded by pharmaceutical, medical device or other health care industry*					
Yes	40	2.61	.83	t = 1.06	.30
No	260	2.47	.59		
*Human subjects research: social or behavioral*					
Yes	96	2.33	.52	t = 3.31	.001
No	204	2.56	.66		
*Human subjects research: clinical*					
Yes	138	2.45	.66	t = .92	.36
No	162	2.52	.60		
*Animal research*					
Yes	111	2.62	.71	t = −2.97	.003
No	189	2.40	.56		
*Dry Lab*					
Yes	54	2.45	.51	t = .57	.57
No	246	2.49	.65		
*Wet Lab*					
Yes	131	2.63	.68	t = −3.52	.001
No	169	2.38	.56		
*Racial categories: white*					
Yes	235	2.45	.61	t = 1.58	.12
No	65	2.60	.70		
*Native language*					
Native english speaker	252	2.43	.57	t = −3.28	.001
English as a second language*	48	2.75	.83		

* All participants held a PhD and worked in the US. The HIT-Res is written at a Flesch–Kinkaid 5th grade reading level

### Statistical Analysis

Data were analyzed using IBM’s SPSS Statistics edition 22 software. Data analysis focused on producing descriptive statistics, reliability statistics for the HIT-Res, confirmatory factor analysis of the HIT-Res, and testing the convergent validity of the HIT-Res by examining correlations with the PDR, PMD, GCS, and NPI-16.

### Research Ethics

The Institutional Review Board at Washington University in St. Louis approved the study using an expedited protocol (201401153). The survey included a 4-page consent form. Participants indicated consent by clicking a button to proceed to the measures.

## Results

The results pertained to the psychometric properties of the HIT-Res in relation to reliability, demographic group, internal factor structure, and construct validity.

### HIT-Res Reliability

Nine cognitive distortion (CD) items and one anomalous responding (AR) item had a corrected item-total correlation less than .38 and were dropped from further analyses. Subsequent analyses focused on the 45-item version of the HIT-Res presented in “Appendix”, which consists of 33 CD items—8 assuming the worst (AW), 9 blaming others (BO), 8 minimizing/mislabeling, and 8 self-centered (SC) items—as well as 6 AR and 6 positive filler (PF) items. The remaining items have an item-total correlation range from .38 to .64.

Cronbach’s alpha reliability coefficients were .92 for the 33 CD items and .75 for the AR scale. (PF is not a scale and therefore reliability scores were not generated.) These alphas are nearly identical to those observed in the meta-analysis of studies conducted with the original HIT (.93 and .72, respectively).

All four CD subscales (AW, BO, MM, and SC) were correlated with each other in the range of *r* = .69 to .88 (*p* < .001) and with the HIT-Res total score in the range of *r* = .85 to .89 (*p* < .001). This raised the question of whether the subscales are in fact separate factors; however, such strong correlations were also observed in the original HIT (Barriga et al. 2001).

### Distribution and Demographic Differences

**Fig. 1:**
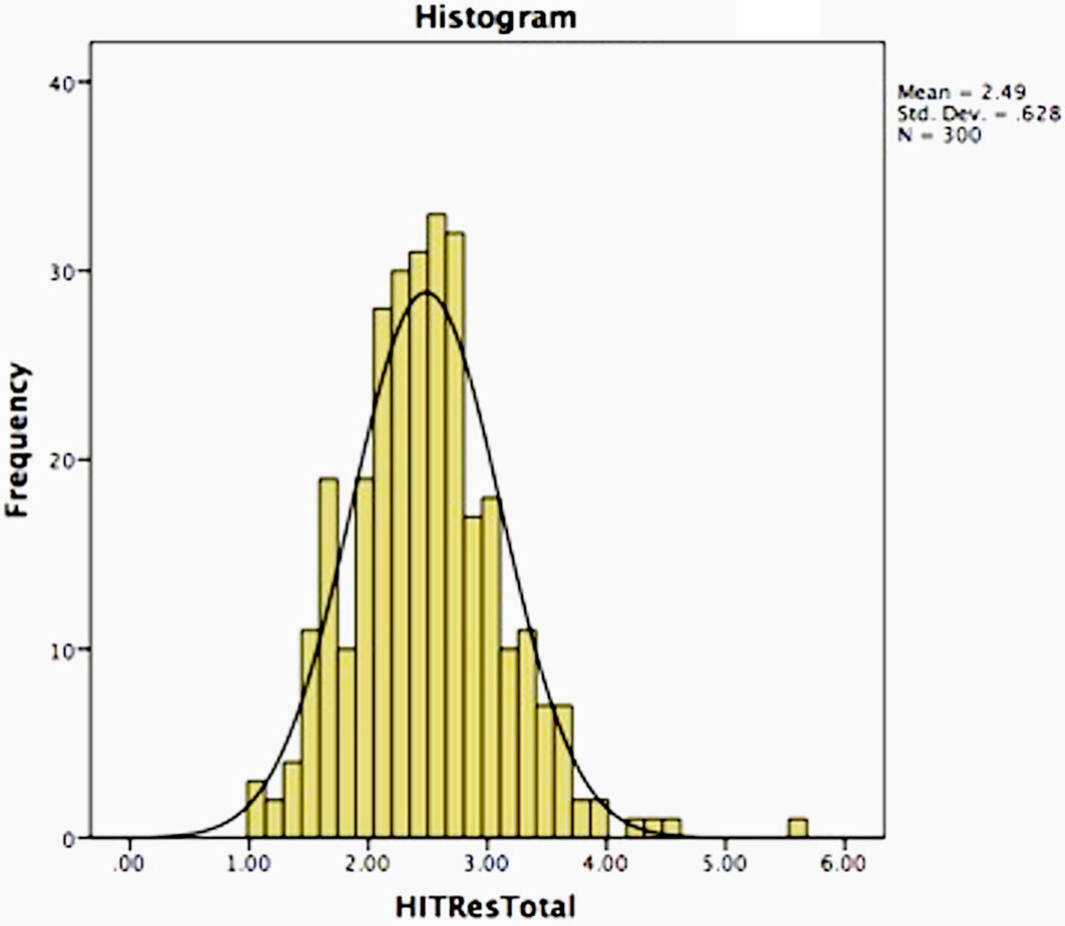
Distribution of HIT-Res mean scores

The overall sample (*N* = 300) had a mean HIT-Res score of 2.49 (SD = .63) with a range of 1.06–5.55. While the HIT-Res is not the same test as the HIT, the mean score observed among control populations (non-offenders, *N* = 3676) in a meta-analysis of original HIT data is nearly identical to what we observed (*m* = 2.47). Figure 1 indicates the distribution of HIT-Res scores, which resembles closely the bell curve typical of normally distributed traits.

Table 2 presents demographic data, including mean scores and tests of significant differences (*t* tests and ANOVAs) between groups. Higher scores are associated with being male (*t* = 3.42, *p* < .001), an animal researcher (*t* = 2.97, *p* = .003) or wet lab researcher (*t* = 3.52, *p* < .001), and having English as a second language (*t* = 3.28, *p* < .001). No significant differences were associated with years of experience, status as a trainee versus independent investigator, or with race (once English as a second language was taken into account).

### HIT-Res Confirmatory Factor Analysis

Past studies of the original HIT have used confirmatory factor analysis to test the fit of two different models: A six-factor model that treats each of four cognitive distortions as unique factors, plus the AR scale, and PF items; and a three-factor model that treats all of the four cognitive distortions as one factor, plus the AR scale, and PF. Barriga et al. (2001) found a better fit using the six-factor model, while Wallinius et al. (2011) found a better fit with the three-factor model. Accordingly, we ran both six-factor and three-factor models using confirmatory factor analysis.

Maximum likelihood estimation confirmatory factor analysis (IBM SPSS AMOS 22.0.0, Amos Development Corporation, Meadville, PA) was used to assess the structure of the HIT-Res. Model fit was evaluated using the Chi squared test, ratio of the Chi squared value to the model degrees of freedom (df), root mean square error of approximation (RMSEA), goodness-of-fit index (GFI), and parsimony goodness-of-fit index (PGFI). Standardized path coefficients were estimated (with standard errors) for all models.

A six-factor model was tested first. This model characterized the cognitive distortion construct by the original four dimensions of BO, AW, SC, and MM, and also included AR and PF. The confirmatory model was not admissible, secondary to a non-positive definite covariance matrix and negative variance estimates. A three-factor model treated the cognitive distortion items as one factor, again including AR and PF. This model demonstrated adequate fit: N = 300, χ^2^ = 1897, *df* = 942, *p* < .001; χ^2^/*df* = 2; RMSEA = .058, GFI = .77, PGFI = .70. Table 3 displays the standardized coefficients for the three-factor model and the internal consistency reliability (coefficient alpha) for the CD and AR factors. Correlations among the three factors were: CD-AR, *r* = −.50 (*p* < .001); CD-PF, *r* = −.30 (*p* < .001); and AR-PF *r* = .13 (*p* = .02).

**Table 3:** Standardized path coefficients (error terms in parentheses) and internal consistency reliabilities (α) for confirmatory three-factor model of the HIT-Res

HIT-Res items	Cognitive distortion (α = .92)	Anomalous responding (α = .75)	Positive filler
2-AR		.59 (.35)	
12-AR		.36 (.13)	
15-AR		.81 (.66)	
24-AR		.71 (.51)	
27-AR		.51 (.26)	
28-AR		.53 (.28)	
4-PF			.47 (.22)
23-PF			.42 (.17)
39-PF			.51 (.26)
41-PF			.18 (.03)
43-PF			.39 (.15)
44-PF			.44 (.19)
1-CD (BO)	.54 (.30)		
6-CD (BO)	.57 (.32)		
10-CD (BO)	.47 (.22)		
16-CD (BO)	.55 (.31)		
17-CD (BO)	.49 (.24)		
22-CD (BO)	.42 (.18)		
26-CD (BO)	.52 (.27)		
30-CD (BO)	.52 (.27)		
33-CD (BO)	.67 (.45)		
3-CD (AW)	.50 (.25)		
5-CD (AW)	.48 (.23)		
7-CD (AW)	.52 (.27)		
8-CD (AW)	.47 (.22)		
19-CD (AW)	.54 (.29)		
20-CD (AW)	.39 (.15)		
31-CD (AW)	.41 (.17)		
42-CD (AW)	.57 (.33)		
9-CD (SC)	.55 (.30)		
11-CD (SC)	.52 (.27)		
14-CD (SC)	.46 (.21)		
18-CD (SC)	.51 (.26)		
21-CD (SC)	.41 (.17)		
25-CD (SC)	.66 (.44)		
32-CD (SC)	.55 (.31)		
37-CD (SC)	.44 (.19)		
13-CD (MM)	.50 (.25)		
29-CD (MM)	.38 (.14)		
34-CD (MM)	.55 (.30)		
35-CD (MM)	.55 (.30)		
36-CD (MM)	.49 (.24)		
38-CD (MM)	.65 (.42)		
40-CD (MM)	.59 (.35)		
45-CD (MM)	.58 (.34)		

*AR* anomalous responding, *PF* positive filler, *CD* cognitive distortion, *BO* blaming others, *AW* assuming the worst, *SC* self-centered, *MM* minimizing/mislabeling

### HIT-Res Validity

Table 4 reports the correlation of the HIT-Res with various measures of convergent and concurrent validity. The HIT-Res was strongly correlated with the PMD scale (*r* = .75, *p* < .001). This is strong evidence of convergent validity; that is, the HIT-Res appears to measure moral disengagement in the context of research compliance. This correlation is much stronger than any of the correlations with other convergent validation measures reported in the meta-analysis of data from 29 independent samples using the original HIT, which ranged from .38 to .55 (Gini and Pozzoli 2013).

**Table 4:** HIT-Res construct validity correlations

Measure	Pearson’s r	*p* value
Moral disengagement (PMD)	.75	<.001
Cynicism (GCS)	.51	<.001
Narcissism (NPI-16)	.10	.09
Professional decision-making in research (PDR)	−.38	<.001
Social desirability (MCSDS)*	.23	<.001

* The HIT-Res contains its own self-serving bias scale—the AR or anomalous responding scale—which positively correlates with the Marlowe–Crown social desirability scale (MCSDS) at .56, *p* < .001. Thus, it has a built in control for social desirability

As expected, the HIT-Res was also positively correlated with the GCS (*r* = .51, *p* < .001). Somewhat surprisingly given that the HIT-Res assesses self-serving cognitive distortions, it was not significantly correlated with the NPI-16 (*r* = .10, *p* = .09). However, this may suggest that the thinking patterns assessed by the HIT-Res and used in moral disengagement are not unique to any personality type.

Overall HIT-Res scores were weakly correlated with social desirability as measured by the MCSDS (*r* = .23, *p* < .001). As expected, the MCSDS was significantly correlated with the AR score (*r* = .56, *p* ≤ .001), thus providing support for the AR scale as a built in measure of social desirability. At the same time, the statistical significance of the various relationships identified above was not affected when we treated MCSDS or AR responding as a covariate.

Regarding concurrent criterion validity, the HIT-Res was negatively correlated with the PDR (*r* = −.38, *p* < .001). This was expected because good professional decision-making involves considering the rules for research, questioning one’s motives and assumptions, and anticipating consequences in a realistic manner. To investigate the incremental validity of the HIT-Res in relation to the PDR, two multiple regression analyses were conducted. In the first analysis, PMD, GCS, NPI-16, and MCSDS were entered as a block to predict PDR. This block explained 14 % of the variance in PDR, with significant regression coefficients associated with PMD (beta = −.27, *p* < .001), GCS (beta = −.15, *p* = .015), and NPI-16 (beta = −.12, *p* = .027); MCSDS was not a significant predictor (beta = −.07, *p* = .20). Following this block, the HIT-Res was entered into the equation. The HIT-Res significantly predicted PDR (beta = −.28, *p* = .001), accounting for an additional 3.2 % of variance explained. With HIT-Res in the equation, the regression coefficients for PMD and GCS were no longer significant (beta = −.09 and −.08, *p* = .28 and .20, respectively). In the second regression analysis, all predictors were considered for inclusion in an equation to predict PDR based on a statistical (stepwise) inclusion rule. In this analysis, HIT-Res entered the equation first, explaining 15 % of the variance in PDR, followed by NPI-16, which explained an additional 1.3 % of the variance. Beta coefficients were −.37 (*p* < .001) for HIT-Res and −.12 (*p* = .03) for NPI-16. PMD and GCS did not enter the equation.

## Discussion

The validity study presented in this article supports the HIT-Res as a valid and reliable adaptation of the original HIT in this population, with psychometric properties (alphas and means) nearly identical to the original HIT. By adapting the behavioral referents from delinquent behaviors to matters of research compliance, we have produced the first measure to assess the use of cognitive distortions regarding research compliance.

Our analysis of the psychometric properties of the HIT-Res did differ from the original HIT in one regard: Whereas the original HIT was determined to be multifactorial—with each of the cognitive distortions functioning as a unique subscale—the HIT-Res clearly represented a single factor for the cognitive distortions. This is not entirely inconsistent with prior data on the original HIT. The HIT manual reported that all cognitive distortions were correlated with each other at .82 or higher. Thus it is questionable whether it was appropriate to run a 6-factor confirmatory factor analysis model. Moreover, Wallinius et al. (2011) also found that the four cognitive distortions in the original HIT functioned as one factor. They speculated that this could be due to population-related factors: Their sample contained adults, whereas the original validity samples for the HIT were comprised of adolescents exclusively. Nevertheless, we believe a one-factor model is theoretically defensible, particularly in light of the strong correlation of the HIT-Res with the Propensity to Morally Disengage scale: A correlation of .75 is typical of parallel forms, suggesting that the two tests tap into essentially the same construct, despite their obviously different referents. At the same time, regression analysis indicated that the HIT-Res retains independent predictive validity, likely because it contextualized to the research setting as is the PDR.

### What Does the HIT-Res Measure?

Here it is worth quoting at length what Moore et al. (2012) wrote about the development of the Propensity to Morally Disengage scale:

Consistent with Bandura’s theoretical claim that moral disengagement is best understood to be “multifaceted” (Bandura et al. 1996: 367), not multifactorial, and in line with both his (e.g., Bandura et al. 1996) as well as subsequent published and unpublished work on moral disengagement…, our aim was to create a unidimensional measure of the general propensity to morally disengage. That is, while acknowledging that the eight individual mechanisms of moral disengagement represent different facets of the construct, our overarching goal was to tap these facets as part of a valid scale that assesses the general propensity to morally disengage as a higher order concept. (p. 13)

Accordingly, the four cognitive distortions can be construed as mechanisms of moral disengagement, and as such represent different facets of a higher order concept. Table 5 illustrates how each of the eight mechanisms of moral disengagement can be mapped onto the four cognitive distortions.

**Table 5:** Mapping the eight mechanisms of moral disengagement onto the four cognitive distortions

Cognitive distortions: (Barriga et al. 2001)	Assuming the worst	Blaming others	Minimizing/mislabeling	Self-centered
Mechanisms of Moral Disengagement: (C. Moore et al. 2012)	Moral justification	Displacement of responsibility; Diffusion of responsibility; Attribution of blame; Dehumanization	Euphemistic labeling; Advantageous comparison; Distortion of consequences	All mechanisms support self-centered thinking by reducing empathy (e.g. dehumanization) or reducing self-sanctioning (Gibbs et al. 1995)

### Practical Applications of the HIT-Res

The HIT-Res may be valuable to Responsible Conduct of Research instructors in at least two ways. First, the HIT-Res provides a new outcome measure—the first measure to examine cognitive distortions in the service of compliance disengagement (Antes and DuBois 2014; Redman 2014). Recent meta-analyses of research ethics courses have found that few courses demonstrate any positive outcomes (Antes et al. 2009, 2010). Although this is explained in part by the instructional methods used, another reason for this finding may be the use of inappropriate outcome measures such as measures of moral development (Antes et al. 2009), which is unlikely to be affected by short-term interventions. In contrast, short-term interventions have been shown to reduce the use of cognitive distortions even in clinically challenging populations (Gibbs et al. 1995).

Second, were courses in the responsible conduct of research to succeed in reducing rates of noncompliance or research misconduct, one would want to know why they had a positive effect. The HIT-Res provides a way of exploring compliance disengagement as a mediating variable in the context of clarification research—that is, research that examines not only whether an educational program is effective in achieving an aim (such as increased compliance) but also why (Cook et al. 2008).

### Limitations and Next Steps

Unlike some measures of moral reasoning, which are difficult to “fake high” (Rest et al. 1999), the HIT-Res is susceptible to socially desirable responding as measured by both the Marlowe–Crowne and by the built in AR scale. However, the relationships between the HIT-Res and cynicism, moral disengagement, and professional decision-making remained even when controlling for socially desirable responding. That is to say, the HIT-Res works as a device for assessing compliance disengagement even when some individuals engage in positive impression management in their responses. Moreover, the HIT-Res has a built-in AR scale, which is strongly positively correlated with the Marlowe–Crowne; we strongly recommend that it be used as a control variable in studies that use the HIT-Res as a correlate or outcome measure.

Second, ours was a convenience sample of 300 NIH-funded researchers. Compared to a limited-variable dataset of over 2500 potential participants in this research, the 300 respondents represented here were more likely to be female (57.3 vs. 40.9 %), χ^2^(1) = 29.1, *p* < .001, and were more likely to be native English speakers (84.0 vs. 77.5 %), χ^2^(1) = 6.5, *p* = .01. The groups were comparable, however, regarding any human subjects research: 60.3 % in the present sample versus 56.2 % in the comparison sample, χ^2^(1) = 1.8, *p* = .178. As a result, to the extent that gender and language are associated with responses, the results obtained here may not be representative of the universe of NIH researchers, though we have no reason to believe that participating female and ESL researchers were different from their non-participating counterparts. Participants were also paid for their time, given the extensive time burden of our testing (approximately 1 h). This may have had the advantage of including a broader and less biased range of participants than we might have had if we relied on altruism alone. To the extent that payments may have created a desire to respond in socially desirable ways, we assessed it using two measures and controlled for it.

In this study, we used a measure of professional decision-making in research as a measure of concurrent validity. While low-fidelity performance simulations have been shown to correlate with actual job performance (Helton-Fauth et al. 2003), it would be desirable to examine directly the relationship of the HIT-Res to job performance, though directly assessing relatively rare and hidden events (such as data fabrication or failure to report conflicts of interest) is challenging. Next steps in our research agenda with the HIT-Res include examining its relationship to *self*-*reports* of violations of research ethics and compliance, though we concede that self-reports are liable to reflect socially desirable responding. Plans for future research also include using the HIT-Res in two analyses of data obtained from participants in the PI Program, which enrolls researchers who have had difficulty with compliance expectations. We wish to examine whether the PI Program, which directly addresses thinking patterns, can effect significant reductions in HIT-Res scores from pre- to post-testing.

We expect that this reduction is possible. After all, Bandura’s theory of moral disengagement was not meant to explain how individuals with antisocial personality could commit atrocities so much as how “normal” people could—in some sectors of their lives at a particular point in history—violate the most basic rules of decent society (Bandura 1999). Perhaps the concept of “compliance disengagement” can accomplish something similar with regard to understanding how “normal” researchers come to violate the basic rules of science.

## Appendix: How I Think About Research (HIT-Res)

Instructions: Each statement in this survey may describe how you think about research. Read each statement carefully then rate the extent to which you agree or disagree with the statement as it describes your current thinking. Your answers will be treated confidentially.

The following abbreviations are used in this questionnaire:

- IRB = Institutional Review Board. (In many nations, these boards are called Research Ethics Committees).
- IACUC = Institutional Animal Care and Use Committee.

1. When IRBs or IACUCs require a lot of changes to research protocols they are just asking for protocol violations.
2. I have done things that I’m not proud of. (AR)
3. You can’t advance a career in research without stepping on someone’s toes.
4. Researchers should strive for excellence in their work. (PF)
5. IRBs and IACUCs focus so much on rules and regulations they can make it impossible to do research.
6. The pressure to get grants almost forces people to take liberties with their data.
7. No matter what I do, someone will find a compliance problem in my research.
8. You cannot expect research collaborators to act with complete integrity.
9. The advancement of my science should have priority over the quality of life of a lab mouse.
10. It’s not my fault if I lose my temper when others produce poor work.
11. I do not have time to deal with IRBs, IACUCs, and other oversight offices.
12. I have sometimes said something bad about a colleague. (AR)
13. Everyone drops data sometimes when they know it’s leading to wrong results.
14. I know which corners I can cut to meet a deadline.
15. I have covered up some things that I have done at work. (AR)
16. My institution makes it too hard to disclose all conflicts of interest.
17. People who don’t understand the realities of animal research are responsible for all of these strict animal care regulations.
18. It’s annoying that institutional committees do not trust researchers to do their jobs right.
19. We can’t be perfect–we just have to muddle our way through when it comes to research ethics.
20. Consent forms don’t protect participants because no one reads them anyway.
21. I really do not need community members or a council to tell me whether my research has social significance.
22. You cannot blame international researchers for plagiarism if they feel forced to publish in English.
23. It’s important to think of colleague’s feelings. (PF)
24. I have misrepresented something to get myself out of trouble. (AR)
25. If I know I’m going to do good science, I really don’t care much about compliance.
26. It is not the principal investigator’s fault when students and lab personnel mismanage animal care.
27. I have tried to get back at someone at work. (AR)
28. In the past, I took something from work without asking. (AR)
29. Courtesy authorship is just part of the culture of research.
30. Journal readers have only themselves to blame if they can’t figure out the bias in industry-funded research.
31. All people prioritize their self-interests whether or not they have conflicts of interest.
32. It’s no one’s business if I make extra money consulting.
33. Sometimes researchers need to deviate from approved protocols to keep study sponsors happy.
34. Most successful researchers bend inclusion criteria a little bit to meet their enrollment goals.
35. No one discloses all of their conflicts of interest.
36. Everybody has conflicts of interest, it’s no big deal.
37. I would require students to complete a survey if it were important to my research.
38. Exaggerating your percent effort on a project is not so bad if the funding is available.
39. When trainees need you, you should be there for them. (PF)
40. Deviating from an approved research protocol is not really a problem if there is no harm to participants, animals, or data.
41. I am generous with my time. (PF)
42. Keeping animal cages clean is so challenging, passing a random inspection is more a matter of luck.
43. I’m happy to share my knowledge with others. (PF)
44. Publications should be open access. (PF)
45. The requirements for animal care are out of proportion to the actual risks to the animals.

Response scale: 1–6 Likert Scale

1. Strongly Disagree
2. Disagree
3. Slightly Disagree
4. Slightly Agree
5. Agree
6. Strongly Agree
